# Approach to Ankle Instability in Patients With a Negative Ankle MRI: A Case Series

**DOI:** 10.7759/cureus.34047

**Published:** 2023-01-21

**Authors:** Waleed K Alnejadi, Ammar Aljefri, Ziyad M Alharbi, Saif Saif, Kenan Nejaim, Mohammed Almutairi, Omar Batouk

**Affiliations:** 1 College of Medicine, King Saud Bin Abdulaziz University for Health Sciences College of Medicine, Jeddah, SAU; 2 Orthopedic, King Saud Bin Abdulaziz University for Health Sciences, Jeddah, SAU; 3 Orthopedic Surgery, King Saud Bin Abdulaziz University for Health Sciences College of Medicine, Jeddah, SAU

**Keywords:** ankle instability, normal mri, posterior talofibular ligament, calcaneo-fibular, anterior talofibular

## Abstract

Objectives

Injuries to the ankle ligaments are some of the most common musculoskeletal sports injuries. Ankle magnetic resonance imaging (MRI) is the standard diagnostic procedure in today’s practice, but its reliability and validity remain controversial. The aim of this study was to explore the approach for patients with negative ankle MRI who continue to have symptoms of ankle instability despite conservative therapy.

Methods

A total of eight patients who were 14 years or older with negative ankle MRI who continue to have symptoms of ankle instability despite conservative therapy were admitted to our institution from January 1, 2015 to December 31, 2021.

Results

Eight patients with a mean age of 36, and a mean body mass index (BMI) of 37.7. All patients presented with ankle pain, locking, and giving way in variable severity. All the patients had a radiograph followed by an ankle MRI, which showed normal alignment of ankle joints without abnormality. Initially, all patients were treated conservatively but did not show any improvement. After that, they underwent an operation of lateral ankle ligament reconstruction by modified Brostrom technique, followed by casting and physiotherapy. The symptoms of ankle instability resolved in all patients. The ankle pain resolved completely in six patients, improved in one patient, and did not improve in one patient.

Conclusion

Based on our results, we advocate reconstruction surgery by modified Brostrom technique for ligament repair in patients with clinical evidence of chronic ankle instability who have failed a trial of conservative management, even in the context of a normal ankle MRI.

## Introduction

Injuries to the ankle ligaments are some of the most common musculoskeletal sports injuries, with lateral ankle ligament injuries accounting for most of all ankle injuries [1,2]. The lateral ankle ligament complex consists of three major ligaments: the anterior talofibular ligament (ATFL), calcaneo-fibular ligament (CFL), and posterior talofibular ligament (PRFL) [3]. The ATFL and the CFL are the most common ligaments to be torn in lateral ligament injuries [1-4]. The PTFL is rarely injured, except in complete dislocation of the ankle [1,4]. Eighty-five percent of ankle sprains involve the lateral collateral ligaments, with a predictable sequence of injury involving the ATFL, followed by the CFL, and finally the PTFL [4].

The evaluation of ankle injuries is based on a history of a forefoot supination/hindfoot inversion injury, clinical signs, and radiological features [1,3]. MRI is a useful tool in diagnosing lateral ligament injuries because it clearly displays abnormalities of injured ligaments, as well as those of adjacent tissues caused by various lesions [3-5]. Although MRI represents a standard diagnostic procedure in today’s practice, its reliability and validity remain controversial [5]. Also, even though most patients are successfully treated conservatively, residual problems may occur, such as chondral lesions of the talus and chronic ankle instability [6]. Chronic ankle instability can be diagnosed clinically by assessing the patient for ankle joint locking and giving way [6].

The initial management of ankle injuries is the RICE protocol, which consists of rest, ice, compression, elevation, and analgesia [7]. Surgical treatment is rare and includes arthroscopy and reconstructive surgeries such as the modified Brostrom technique which is a well-recognized surgical technique used for the treatment of lateral ligamentous complex injuries for ankle instability and is now regarded as the reference standard technique [7,8]. The use of surgical treatment is typically limited to patients who fail conservative management [7,8]. The aim of this study was to explore the approach to patients with negative ankle MRI who continue to have symptoms of ankle instability despite conservative therapy in our institute.

## Materials and methods

A retrospective case series discussed the approach to patients with a negative ankle MRI who continue to have symptoms of ankle instability despite conservative therapy at the Department of Orthopedic Surgery in King Abdulaziz Medical City, Ministry of National Guard Health Affairs, Jeddah.

We included all the patients who were diagnosed with ankle instability symptoms after lateral ankle injury 14 years and older with negative MRI findings. On the other hand, we excluded all the patients who have incomplete charts or requested privacy.

A total of eight patients who are 14 years or older and were admitted to our institution from January 1, 2015 to December 31, 2021 were included. Patients were identified from the NGHA-J database and important variables were extracted from the database. Missing information was extracted from the patient’s charts.

## Results

Case 1

A 38-year-old male soldier in the navy presented to the orthopedic clinic complaining of moderate left ankle pain that started after falling down the stairs seven years before the presentation. He had a height of 168 cm and a Body Mass Index (BMI) of 36.49, and he is a known smoker. On clinical assessment, ankle joint locking, and ankle joint giving way were the significant findings. A radiograph was ordered and found to be normal. This was followed by an MRI that showed normal alignment of the ankle and subtalar articulations. Then, we proceeded with a trial of conservative management with subsequent serial casting for six weeks, Non-Steroidal Anti-Inflammatory Drugs (NSAIDs) for analgesia, and rehabilitation exercises to strengthen and stretch the injured ankle with no improvement for eight months. Surgical treatment was discussed with the patient, and the surgery was scheduled two years after the initial presentation. Intraoperatively, laxity was found in the ATFL and the CFL, and both were repaired by the modified Brostrom technique. Postoperatively, a below-the-knee cast was applied for six weeks, and the patient was instructed to follow up in the clinic. Postoperative assessment in the next one year through showed that the symptoms of pain and joint locking and giving way had resolved.

Case 2

A 28-year-old female presented to the orthopedic clinic with severe right ankle pain that started after falling. She had a height of 160 cm and a BMI of 14.8. She was a non-smoker and had no significant medical history. Upon physical examination, her pain was found to be most severe over the ATFL origin. Further assessment revealed symptoms of ankle instability, locking, and giving way. After that, a trial of conservative management with subsequent serial casting for six weeks was followed by five months of physiotherapy for exercises to strengthen and stretch the injured ankle with minimal improvement and NSAIDs for analgesia. As a result, an MRI was ordered and showed no evidence of a ligamentous tear. Due to the positive clinical findings and failure of conservative treatment, surgery was booked a year after the initial presentation for reconstruction of the lateral ankle ligament. Intraoperatively, an ATFL tear was seen and repaired by modified Brostrom technique. Postoperatively, a below-the-knee cast was applied for six weeks. Postoperative assessment in the next one year showed that the symptoms of ankle pain, joint locking, and giving way had resolved.

Case 3

A 27-year-old female housewife presented to the orthopedic clinic with severe left ankle pain that started after a twisting injury nine months prior to presentation. At the time of the injury, she went to the emergency room and was clinically diagnosed with an ankle sprain. She had a height of 170 cm and a BMI of 29.48, and she was a non-smoker. Her only previous surgery was a tonsillectomy, and she had no significant medical history. On clinical assessment, she had severe ankle pain and ankle joint locking and giving way. She began on a trial of conservative treatment, with serial casting for six weeks, followed by physical therapy for exercises to strengthen and stretch the injured ankle for seven sessions in five months and NSAIDs for analgesia, with no significant improvement. A radiograph was performed and was normal. This was followed by an MRI that showed normal alignment and no evidence of ligament tear. Due to the positive clinical findings and failure of conservative treatment, surgery was booked for exploration of the lateral side of the left ankle and lateral ankle ligament reconstruction. Intraoperatively, there was evidence of laxity in both the ATFL and CFL, so reconstruction of the CFL, ATFL, and PTFL was performed by modified Brostrom technique. After the surgery, a cast was applied to the ankle in dorsiflexion and eversion, and the patient was referred for physical therapy. Postoperative assessment in the next one year showed that the symptoms improved dramatically, with complete resolution of the signs of ankle instability and pain. 

Case 4

A 36-year-old male soldier presented to the orthopedic clinic with left ankle pain for two years following a twisting injury. He had a height of 170 cm, a BMI of 36, was an ex-smoker and had no significant medical history. On clinical assessment, he was found to have moderate ankle pain and ankle joint locking and giving way. He began a trial of conservative treatment, which consisted of serial casting for four weeks, NSAIDs for analgesia, and physiotherapy for exercises to strengthen and stretch the injured ankle for five months, but his symptoms did not improve. As a result, an MRI was ordered and showed no evidence of ligament tear. However, as the pain persisted, surgery was booked for left lateral ankle ligament repair. Intraoperatively, there was evidence of laxity in both the ATFL and CFL, so the reconstruction of the CFL and ATFL was performed by the modified Brostrom technique. After surgery, the patient was referred to physical therapy, and casting was applied for four weeks. Postoperative assessment in the next one year showed that the symptoms of pain, joint locking, and giving way had resolved.

Case 5

**Figure 1:**
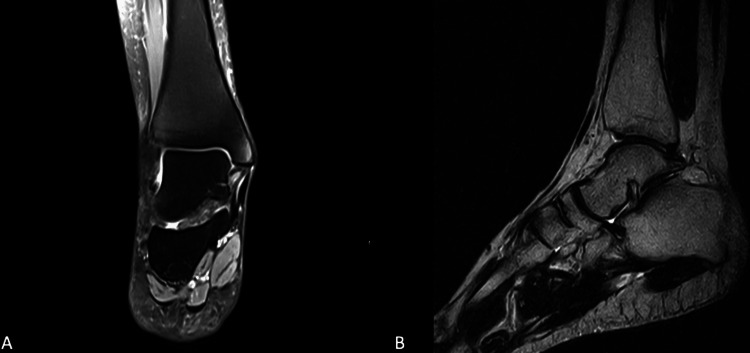
(A, B) MRI images for a 29-year-old male with chronic ankle pain and instability. Radiological diagnosis showed preservation of normal anatomy.

A 29-year-old male soldier presented to the orthopedic clinic complaining of left ankle pain that started one year prior while he was playing football. He had a height of 158 cm and a BMI of 33.3. He is a current smoker and had a medical history of hypertension and pericarditis. On clinical assessment, he was found to have moderate ankle pain and ankle joint locking and giving way. He was started on a trial of conservative treatment, which consisted of serial casting for six weeks, NSAIDs for analgesia, and physiotherapy for exercises to strengthen and stretch the injured ankle for six months, but his symptoms showed no improvement. Consequently, an MRI was ordered, which showed no ligament or tendon injuries. However, degenerative subchondral cystic changes were found in the distal tibia and talar dome. As his pain and instability symptoms persisted, surgery was booked for left lateral ankle ligament repair. Intraoperatively, there was evidence of laxity in both the ATFL and CFL, so the reconstruction of the CFL and ATFL was performed by the modified Brostrom technique. After the surgery, a cast was applied below the knee for the left lower limb, and the patient was referred for physiotherapy. Postoperative assessment in the next one year showed that the patient’s ankle pain and instability had resolved (Figures 1A, 1B).

Case 6

A 36-year-old male soldier presented to the orthopedic clinic complaining of right ankle pain that started several months prior after a twisting injury. He has a height of 178 cm and a BMI of 20.6. He is a known smoker and has no significant medical history. On clinical assessment, he was found to have moderate right ankle pain and ankle joint locking and giving way. The patient reported that the pain increased with running and walking. The patient was treated conservatively by serial casting for four weeks, NSAIDs for analgesia, and physiotherapy for exercises to strengthen and stretch the injured ankle for four months. However, his symptoms did not improve. An MRI was ordered, and it showed no evidence of a ligament tear. As the patient’s symptoms persisted despite conservative therapy, surgery was booked for right lateral ankle ligament repair. Intraoperatively, there was evidence of laxity in both the ATFL and CFL, so the reconstruction of the CFL and ATFL was performed by the modified Brostrom technique. After the surgery, a cast was applied, and the patient was referred for physiotherapy. Postoperative assessment in the next one year showed resolution of the symptoms of ankle instability with some degree of pain improvement, but unfortunately, the pain persisted.

Case 7

A 53-year-old female presented to the orthopedic clinic complaining of right ankle pain with walking that started several months prior. She had a height of 156 and a BMI of 38.4. She was a non-smoker and had a history of hypothyroidism. On clinical assessment, she was found to have mild ankle pain with walking, and ankle joint locking and giving way. A radiograph was ordered and found to be normal. After that, an MRI was ordered, and the report showed a normal image. She tried conservative treatment by serial casting for four weeks, NSAIDs for analgesia, and physiotherapy for exercises to strengthen and stretch the injured ankle for four months, but there was no improvement in her symptoms. Therefore, surgery was booked for lateral ankle ligament repair. Intraoperatively, there was evidence of laxity in both the ATFL and CFL, so the reconstruction of the CFL, ATFL, and PTFL was performed by modified Brostrom technique. Postoperatively, a cast was applied below the knee for four weeks, and the patient was referred for physiotherapy. Postoperative assessment in the next one year showed resolution of the symptoms of ankle instability, but unfortunately, the pain persisted.

Case 8

**Figure 2:**
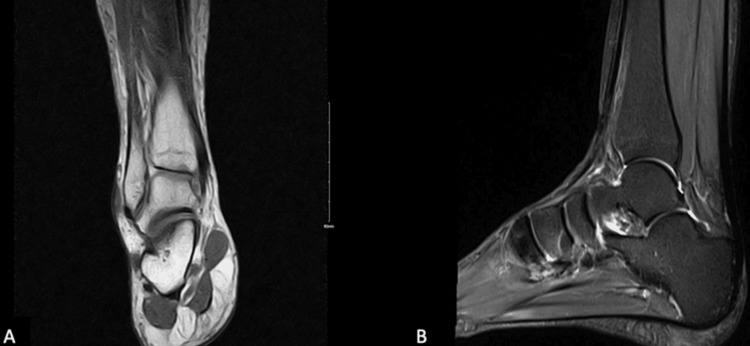
(A, B) MRI images for a 41-year-old male with chronic ankle pain and instability. Radiological diagnosis showed preservation of normal anatomy.

A 41-year-old male presented to the orthopedic clinic complaining of right ankle pain that started one year prior. He had a height of 176 cm and a BMI of 37.1. He was a non-smoker and had no significant medical history. On clinical assessment, he was found to have mild ankle pain, and ankle joint locking and giving way. An MRI was ordered and revealed normal imaging. We tried conservative treatment by serial casting for four weeks, NSAIDs for analgesia, and physiotherapy for exercises to strengthen and stretch the injured ankle for four months, but there with no improvement. Surgery was booked for lateral ankle ligament repair. Intraoperatively, there was evidence of laxity in both the ATFL and CFL, so the reconstruction of the CFL and ATFL was performed by modified Brostrom technique. Postoperatively, a cast was applied below the knee for four weeks, and the patient was referred for physiotherapy. Postoperative assessment in the next one year showed that the symptoms of pain and joint locking and giving way had resolved (Figures 2A, 2B). All cases are summarized in Table 1.

**Table 1:** Description of patients with ankle instability who underwent surgery with normal MRI

SN	Age	Gender	BMI	Smoking	Date of Surgery	Pre-Op Pain	Pre-Op Locking	Pre-Op Giving Way	Post-Op Pain	Post-Op Locking	Post-Op Giving Way	MRI Report
1	38	Male	36.49	Current	30/09/2018	Moderate	Yes	Yes	No	No	No	Normal MRI
2	28	Female	14.8	No	18/08/2019	Sever	Yes	Yes	No	No	No	Normal MRI
3	27	Female	29.48	No	17/06/2019	Mild	Yes	Yes	No	No	No	Normal MRI
4	36	Male	37.1	No	23/07/2016	Mild	Yes	Yes	No	No	No	Normal MRI
5	29	Male	33.3	Current	26/02/2018	Moderate	Yes	Yes	No	No	No	Normal MRI
6	36	Male	20.6	Current	10/04/2021	Moderate	Yes	Yes	Mild	No	No	Normal MRI
7	53	Female	38.36	No	01/01/2018	Mild	Yes	Yes	Moderate	No	No	Normal MRI
8	41	Male	37.1	No	23/07/2016	Mild	Yes	Yes	No	No	No	Normal MRI

## Discussion

The diagnosis of lateral ankle ligament injury can be suspected after a thorough history and physical examination [9,10]. The diagnosis can be confirmed with an MRI showing evidence of damage to the lateral ankle ligament. In our case series, we reported eight cases that returned a normal MRI but were later confirmed to have lateral ankle ligament injuries during surgery. Also, these findings are consistent with the literature, as studies have shown that MRI has a sensitivity of 75% to 96% in the diagnosis of high-grade injuries to the lateral ankle ligament [4,10]. It is because of this that we warn against using MRI to rule out lateral ankle ligament tears. In our study, the median BMI was 31, with five patients over 30 years old. Moreover, according to Vuurberg, a high BMI may be considered a risk factor for lateral ankle instability [11].

The initial treatment for lateral ankle ligament injuries is conservative therapy, which focuses on improving strength and muscle reaction time. The most widely used conservative therapies are physical therapy, taping, and bracing, which can be applied to any patient with ankle instability [12]. However, some studies have reported that patients who receive conservative therapy still complain of persistent ankle pain, functional instability, and swelling [12]. In our study, all eight cases started with conservative therapy, including casting for an average of four to six weeks, physiotherapy for an average of four to six months, and analgesia, but without any improvement. Scott et al. also found that surgical treatment in ATFL is more effective than conservative treatment [13].

The most common indications of surgical management are mechanical and functional instability [12]. Surgery helps in the dynamic assessment of joint instability, which can be difficult with MRI [4]. In our study, the median time of waiting from the initial presentation and surgery was one year, which indicates the failure of conservative therapy. As a result, all eight cases underwent reconstruction surgery by modified Brostrom technique. All cases showed that the symptoms of joint locking and giving way had resolved, and the ankle pain resolved in six out of eight patients following surgery, despite the preoperative negative ankle MRI results. Moreover, these findings are consistent with the literature, as studies have shown that surgical intervention for CAI has a better outcome for pain and joint instability [8,14].

There were some limitations in our study which include the difficulties in data collection in retrospective studies, such as the details of the patients’ history prior to presenting to the orthopedic clinic. While our patients were assessed clinically for signs of instability, no objective scoring system was used to describe this assessment.

## Conclusions

In this study, we performed surgical reconstruction by modified Brostrom technique for patients who complained of symptoms of chronic ankle instability and failed conservative management despite negative MRI results. Intraoperatively, all of our patients were found to have evidence of ligamentous laxity. All of the patients who underwent the procedure reported improvement in their symptoms of instability, and six out of eight patients reported that their pain went away. Therefore, we advocate for our colleagues to attempt surgical reconstruction in patients suffering from chronic ankle instability who do not respond to conservative management even in the absence of positive MRI findings.
